# Supplementary figures and images for: Stress responses of the oil-producing green microalga Botryococcus braunii Race B
Source: PeerJ. 2016 Dec 6;4:e2748. doi: 10.7717/peerj.2748 (PMC5144741; doi:10.7717/peerj.2748)

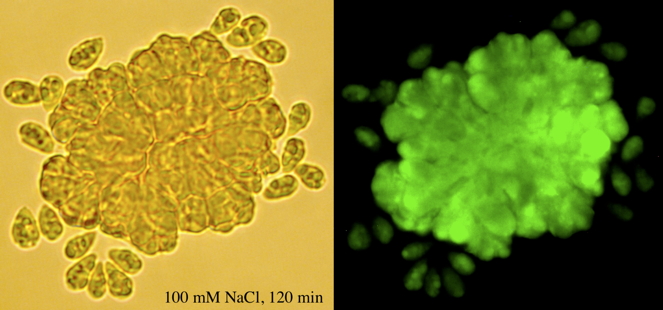

Supplement: Figure S2 — B. braunii colonies after 120 min or treated with higher concentrations of inducers were damaged. The colonies were disintegrating and unspecific fluorescence was present most probably due to carotenoids produced by damaged cells ((Davis et al., 2014) Algal Research 5: 181–189; (Morosinotto & Bassi , 2014) Advances in Photosynthesis and Respiration, pp. 315–331). [file peerj-04-2748-s007.jpg]

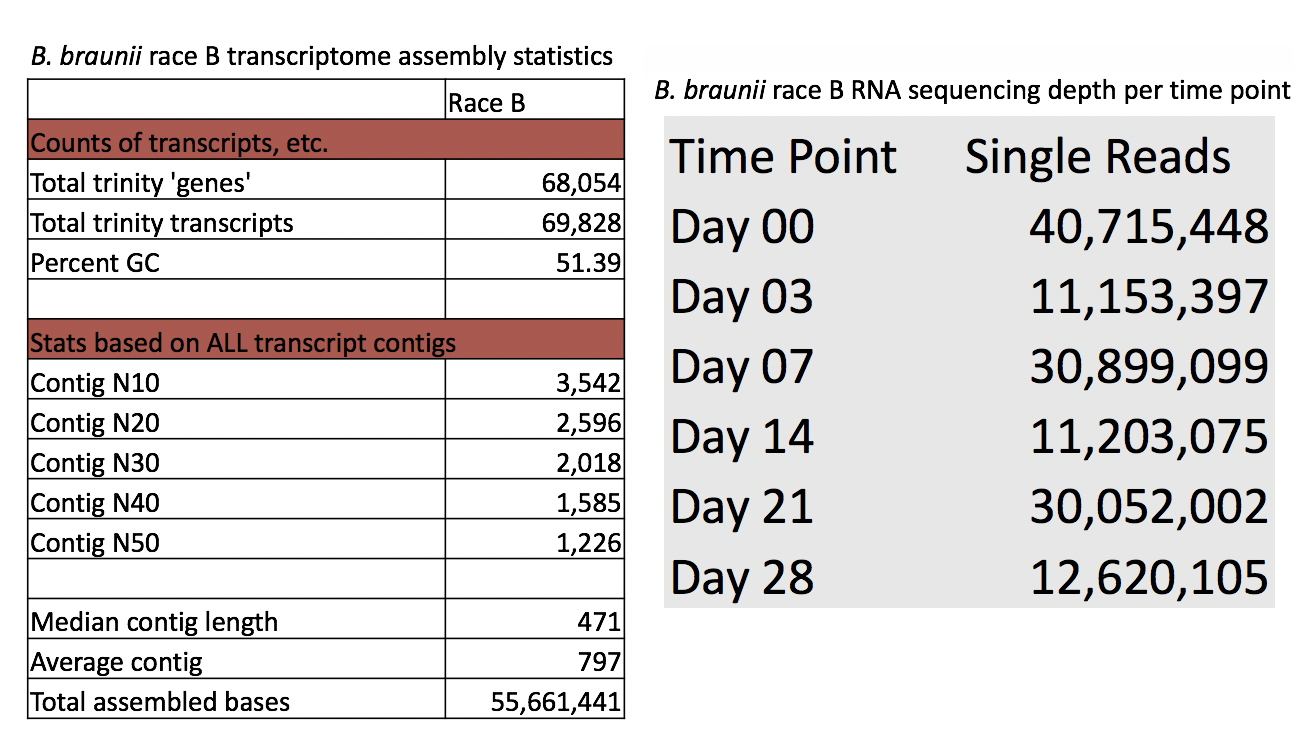

Supplement: Figure S3 — Analysis of the data on the assembly such as number of contigs, N50, and total number of sequence reads at each data point is also provided. [file peerj-04-2748-s008.jpg]
